# Inquiries Concerning Anæsthetics

**Published:** 1878-05

**Authors:** 


### ARTICLE VII.

### *Inquiries Concerning Anæsthetics.*

At its last meeting the British Medical Association appointed a committee to make further studies on anæsthesia, especially with regard to the relative merits of chloroform, ether and bichloride of methylene. The last mentioned, by the by, is preferred to either of the former by Mr. F. Spencer Wells and other eminent British surgeons.

Such committees are much needed, for there are a great many points about anæsthetics not yet satisfactorily settled. Some of them are of great medico legal importance, and considering how intimately they concern medical men, even to the extent of putting their personal and professional reputation in peril every time they administer an anæsthetic to a female without competent and truthful witnesses standing by, it is extraordinary that greater attention has not been given them.

Quite lately a medical man in England was arrested and imprisoned on the charge of a married woman that he had violated her after giving her chloroform to extract a tooth. She claimed that though wholly speechless and powerless, from the anæsthetic, and therefore unable to resist, her mind was quite awake and she knew everything that took place. A gentleman on the trial deposed to a similar occurrence in the experience of a friend.

Here, therefore, is a question we would ask, and one obviously of vital importance. If this condition of complete consciousness does at times coexist with complete motor and sensory anæsthesia, of course the plea of imagination offered in such cases as the usual defence falls to the ground. For our own part, we have administered both chloroform and ether very frequently, but never knew an instance when the patient was so completely under the influence of the agent that he felt no pain, in which consciousness or memory remained active.

Again, there is the old inquiry as to whether a person in sleep can be anæsthetized.

The latest thorough study of these and kindred questions which we have seen is by Professor R. M. Denig, in the *Ohio Medical Record*, January, 1877 (given in the *Half-Yearly Compendium*, July, 1877.) His general conclusions are that chloroform cannot be used successfully for felonious purposes, and that a person in the anæsthetic state is not a competent witness, which conclusions we believe to be correct.

In regard to ether, there are some inquiries of a different character worthy of attention. We were recently asked by a very observant surgeon whether cases were recorded where a single administration of ether left behind it permanent impairment of the digestive and nervous systems, extending over years. He narrated one such case in his own experience, in the person of a strong and healthy young man, who took the anæsthetic for a trifling operation. Such an instance was known to us, but the subject was a hysterical woman, and we never felt sure how many of her symptoms were imaginary.

More than one death from ether have been lately recorded. Mr. Robert Saundly, of Birmingham, gives the following typical instance in the London *Medical Press and Circular*:—

M. C., aged 35, was admitted for contracted knees. On October 4th, at 12.45 P. M., I administered ether with Ormsby's apparatus; it appeared to me a very favorable case; very little of the anæsthetic was used; there were no alarming incidents; very little stertor or cyanosis; no vomiting; no obstruction to respiration, which was throughout regular and full. After Mr. Bartleet had straightened the limbs, some time was consumed in adjusting splints, during which time no ether was given; and, as there appeared to be absolutely nothing to call for any notice at the time, I watched her with the utmost satisfaction, and allowed her to be carried out of the theatre without arousing her from the sleep into which she had fallen. She was removed on a stretcher, and was well wrapped up, but, to reach her ward, was carried about fifty yards across the open court, the day being fine. After being placed in bed, she roused and spoke to the nurse, who noticed nothing unusual about her. At 2.45, about one hour and a half after her return to the ward, she became suddenly alarmingly ill, and when seen by the house physician (in the absence of the house surgeon) she was cyanotic and pulseless, with *rales* all over the thorax. All attempts to rally her were fruitless, and she died at 4.15 the same afternoon.

The post-mortem examination, made the following day, showed some œdema of the membranes of the brain; no thrombosis of the pulmonary artery; heart healthy, containing a little blood in the right auricle; ventricles contracted; lungs pale and œdematous; other organs healthy.

There seems to be no doubt that the deceased completely recovered from the ether narcosis, but died from œdema of the lungs, which supervened one hour and a half after her removal from the theatre

Whatever the immediate pathological cause of death here, it is to the ether-narcosis that that condition is to be attributed. It illustrates that too confident reliance on ether as a harmless agent is both unfounded and dangerous.—*Editorial in Med. and Surg. Reporter.*

